# Zoledronic Acid Is Not Equally Potent on Osteoclasts Generated From Different Individuals

**DOI:** 10.1002/jbm4.10412

**Published:** 2020-09-29

**Authors:** Anaïs M J Møller, Jean‐Marie Delaisse, Jacob B Olesen, Troels Bechmann, Jonna S Madsen, Kent Søe

**Affiliations:** ^1^ Clinical Cell Biology Lillebaelt Hospital, University Hospital of Southern Denmark Vejle Denmark; ^2^ Department of Regional Health Research University of Southern Denmark Vejle Denmark; ^3^ Clinical Cell Biology, Department of Pathology Odense University Hospital Odense Denmark; ^4^ Department of Clinical Biochemistry and Immunology Lillebaelt Hospital, University Hospital of Southern Denmark Vejle Denmark; ^5^ Department of Clinical Research University of Southern Denmark Odense Denmark; ^6^ Department of Molecular Medicine University of Southern Denmark Odense Denmark; ^7^ Department of Oncology Lillebaelt Hospital, University Hospital of Southern Denmark Vejle Denmark; ^8^ OPEN, Open Patient data Explorative Network University of Southern Denmark Odense Denmark

**Keywords:** OSTEOCLASTS, ANTIRESORPTIVES, TUMOR‐INDUCED BONE DISEASE, OSTEOPOROSIS

## Abstract

Zoledronic acid is a bisphosphonate commonly used to treat bone diseases such as osteoporosis and cancer‐induced bone disease. Patients exhibit a variable sensitivity to zoledronic acid; the underlying explanation for this remains unclear. The objective of this study was to obtain more knowledge in this regard. We hypothesized that osteoclasts generated from different individuals would show a variable sensitivity to zoledronic acid in vitro. Osteoclasts were generated using monocytes from 46 healthy female blood donors (40 to 66 years). Matured osteoclasts were reseeded onto bone slices precoated with different concentrations of zoledronic acid. IC50 values were determined based on total eroded bone surface after 3 days of resorption. The IC50 for inhibition of osteoclastic bone resorption varied from 0.06 to 12.57μM zoledronic acid; thus, a more than 200‐fold difference in sensitivity to zoledronic acid among osteoclasts from different individuals was observed. Multiple linear regression analyses showed that the determined IC50 correlated with smoking status, and the average number of nuclei per osteoclast in vitro. Further analyses showed that: (i) increasing protein levels of mature cathepsin K in osteoclast cultures rendered the osteoclasts less sensitive to zoledronic acid; (ii) surprisingly, neither the gene nor the protein expression of farnesyl diphosphate synthase was found to correlate with the IC50; and (iii) trench‐forming osteoclasts were found to be more sensitive to zoledronic acid than pit‐forming osteoclasts within the same cell culture. Thus, we conclude that there indeed is a high degree of variation in the potency of zoledronic acid on osteoclasts when generated from different individuals. We propose that our findings can explain some of the varying clinical efficacy of zoledronic acid therapy observed in patients, and may therefore be of clinical importance, which should be investigated in a clinical trial combining in vitro and in vivo investigations. © 2020 The Authors. *JBMR Plus* published by Wiley Periodicals LLC on behalf of American Society for Bone and Mineral Research.

## Introduction

Antiresorptive drugs, such as bisphosphonates and denosumab, are commonly used in the treatment of bone diseases, including osteoporosis, metastatic cancer‐induced bone disease, and the hematological cancer—multiple myeloma.^(^ ^1^, ^2^ ^)^ One of the most potent bisphosphonates is zoledronic acid (Zol; also called zoledronate), which has been used for almost two decades to treat bone disease.^(^ ^3^, ^4^ ^)^ However, a substantial variation in the effectiveness of treatment has been observed in patients.^(^ ^5^, ^6^, ^7^, ^8^, ^9^ ^)^ Although this variation in the potency of Zol treatment has also been observed in osteoporosis,^(^ ^8^, ^9^ ^)^ it has been shown more clearly in patients with breast cancer and bone metastases.^(^ ^5^, ^6^, ^7^ ^)^

Cancer cells located in the bone marrow induce bone disease by interacting with the local bone cells and the cells of the bone marrow.^(^ ^10^ ^)^ The cancer cells produce factors that directly or indirectly facilitate the local generation and activity of bone‐resorbing osteoclasts (OCs).^(^ ^10^ ^)^ Bone is a lavish reservoir of inactive growth factors that are activated and/or released during the bone‐resorptive process, thereby stimulating the growth of tumor cells.^(^ ^10^ ^)^ In this way, cancer cells and OCs end up engaging in a self‐perpetuating cycle.^(^ ^10^, ^11^ ^)^ As part of the standard treatment for the bone disease, patients with breast cancer are therefore treated with an antiresorptive drug to target OCs and their bone‐destructive activity in an attempt to break the vicious cycle.

However, despite Zol's well‐documented protective effect on many patients, a substantial number of patients still develop new skeletal‐related events (SREs). In fact, a series of studies has shown that up to 50% of patients with breast cancer and bone metastases who receive monthly bisphosphonate treatment still develop new SREs within 1 year of starting treatment, and up to 65% within 2 years.^(^ ^5^, ^6^ ^)^ In comparison, up to 70% of patients with breast cancer receiving no antiresorptive treatment develop new SREs within 1 year; thus, not all patients benefit from the treatment.^(^ ^5^, ^6^ ^)^ Similar observations have been made for denosumab.^(^ ^5^, ^12^ ^)^ With breast cancer being one of the leading causes of cancer‐related mortality in women,^(^ ^13^ ^)^ and bone being the most frequent site of metastasis for patients with breast cancer,^(^ ^11^ ^)^ strategies to reduce both the incidence and morbidity of these bone metastases are evidently of clinical importance.

Bisphosphonates are drugs used to target OCs.^(^ ^2^, ^14^ ^)^ They have a strong affinity for hydroxyapatite and are—through the adsorption to mineralized bone surfaces—eventually absorbed by OCs during bone resorption, ultimately resulting in compromised resorptive activity and possibly apoptosis.^(^ ^14^, ^15^ ^)^ Following bisphosphonate uptake, OCs demonstrate changes in characteristic morphological features, such as a lack of a ruffled border and disruption of the cytoskeleton, as well as vesicular trafficking.^(^ ^14^, ^15^, ^16^ ^)^ Zol is reported to inhibit enzymes of the mevalonate/cholesterol biosynthetic pathway.^(^ ^14^, ^17^ ^)^ Enzymes in this pathway metabolize pyrophosphate‐containing isoprenoid lipids, including isopentenyldiphosphate, farnesyl diphosphate, and geranylgeranyl diphosphate. These lipids are necessary for posttranslational modification (prenylation) of small GTPases.^(^ ^18^, ^19^, ^20^ ^)^ Small GTPases are important signaling proteins in the regulation of several important OC functions, such as cell morphology, cytoskeletal arrangement, membrane ruffling, trafficking of vesicles, and apoptosis.^(^ ^21^, ^22^ ^)^ Prenylation is required for proper subcellular protein trafficking, as the lipid prenyl group serves to anchor the proteins in the cell membrane.^(^ ^23^ ^)^ Inhibition of the mevalonate/cholesterol biosynthetic pathway may also lead to intracellular accumulation of metabolites, such as isopentenyl pyrophosphate and dimethylallyl pyrophosphate. This accumulation may alter cell signaling and induce apoptosis.^(^ ^24^ ^)^

Although the use of more effective anticancer treatments has improved survival,^(^ ^25^, ^26^ ^)^ the ability of antiresorptive agents to suppress new SREs has remained roughly unchanged over the past decades.^(^ ^5^, ^6^, ^27^ ^)^ Patients generally have poorer prognosis and shorter overall survival when the pathological bone turnover is not fully suppressed.^(^ ^7^, ^28^, ^29^ ^)^ Lipton and colleagues evaluated the association of changes in bone turnover markers following denosumab or Zol treatment with overall survival, disease progression, and disease progression in bone in patients with advanced cancer and bone metastases.^(^ ^7^ ^)^ They reported that a low sensitivity (bone turnover marker levels ≥ median) to antiresorptive treatment after 3 months was associated with reduced survival, increased risk of disease progression, and increased risk of disease progression in bone.^(^ ^7^ ^)^ In addition, patients with SREs in general experience a reduced quality of life, and consume significantly more health resources compared with patients without SREs.^(^ ^27^, ^30^ ^)^ Therefore, it is relevant to search for the mechanisms responsible for the incomplete suppression by Zol^(^ ^6^ ^)^ to enable targeted therapy with the overall goal to improve survival and quality of life. The reason for the incomplete suppression of bone resorption is, without a doubt, multifactorial. However, we speculate that one reason may be that patients are not identical, and that their OCs therefore respond differently to the same treatment. Hence, we hypothesized that human OCs generated from different individuals will show a variable sensitivity to Zol in vitro, and that intrinsic differences between individuals can explain this variation.

## Participants and Methods

### Study approval

The protocol was approved by the scientific ethical committee for the region of Southern Denmark with approval number S‐20150059. Written informed consent was obtained from participants before inclusion, and all participants were pseudoanonymized during the study.

### Demographics and sample collection

Forty‐six healthy female blood donors (between 40 to 66 years of age) were recruited from the blood donor corps at Lillebaelt Hospital, University Hospital of Southern Denmark. Demographic characteristics of the donors are given in Table 1. Exclusion criteria were: (i) prior bisphosphonate treatment, and (ii) fractures within the last 2 years. A regular 500‐mL blood donation was obtained and fractioned, and the buffy coat was collected for further use. In addition, 4‐mL venous blood was drawn in the fasting state approximately 2 weeks (mean: 12.8, median: 14 days) after blood donation. To obtain serum, the blood was allowed to clot at room temperature for 30 minutes, before the samples were centrifuged at 2000*g* for 10 minutes. Then, the serum phase was transferred into cryotubes and stored at −80°C. Using questionnaires, information about lifestyle and medical history was provided. Demographics of the donor population are presented in Table 1. As participants were collected from the existing pool of blood donors, they were all considered healthy, although six of the donors had minor medical conditions, specified in Table 1.

**Table 1:** Demographic Characteristics of 46 Female Blood Donors

	Clinical features	*n* = 46	%
Age (years)	40–44	8	17.4
	45–49	6	13.0
	50–54	13	28.3
	55–59	10	21.7
	60–67	9	19.6
Menopause status	Premenopausal	16	34.8
	Postmenopausal	30	65.2
Smoking status	Nonsmoker	40	87.0
	Smoker	6	13.0
Comorbidity	No	40	87.0
	Yes	6	13.0
	Hypothyroidism	2	
	Asthma/allergy	3	
	Ulcers	1	
	Mean (SD) [range]	*n*	
Age (years)	53.0 (6.87) [40–66]	46	
Height (m)	1.70 (0.06) [1.56–1.84]	46	
Weight (Kg)	73.2 (13.6) [55.0–124.0]	46	
BMI	25.4 (4.1) [19.5–37.8]	46	

The described donor population is a subset of the donor population that has been reported on previously.^(^ ^46^ ^)^ BMI = Body mass index.

### In vitro generation of human OCs

CD14^+^ monocytes were isolated from buffy coats separately and differentiated to mature OCs.^(^ ^31^ ^)^ Briefly, CD14^+^ cells were purified from peripheral blood mononuclear cells using antihuman CD14 magnetic particles (BD Biosciences, San Jose, CA, USA). Cells were seeded at a density of 5 × 10^6^ cells in T75 culture flasks and differentiated into mature OCs (2 days with M‐CSF followed by 7 days with M‐CSF and RANKL; R&D Systems, Abingdon, UK) as previously described.^(^ ^31^, ^32^, ^33^ ^)^ At this point, 12 systematic and evenly distributed pictures of the OCs were taken using a CKX41 microscope with an SC30 camera (Olympus Corp., Tokyo, Japan). The number of OCs with ≥2 nuclei^(^ ^31^ ^)^ and the mean number of nuclei per OC were manually quantified.

### Coating of bone slices with Zol

A test was performed to determine the fraction of Zol binding to cortical bone slices. Then, 200‐μL α‐modified essential medium (α‐MEM) with fluorescently labeled Zol (5‐FAM‐ZOL; BioWinc, Pasadena, CA, USA**)** was added at five different concentrations (0, 3, 10, 30, and 50μM) to the wells of a 96‐well plate, with or without a cortical bovine bone slice (Boneslices.com, Jelling, Denmark; *n* = 5). After 24 hours of incubation at 37°C in the dark, the fluorescence was read at 493 to 521 nm (Synergy HTX multimode reader; Biotek Instruments, Winooski, VT, USA). Results revealed that on average, 94.21% (SD = 2.97; data not shown) of Zol had bound to the bone slice, showing that different concentrations of Zol bind equally well to bone slices. Zol (Fresenius Kabi AB, Uppsala, Sweden) was diluted to the following eight concentrations; 0, 0.03, 0.1, 0.3, 1, 3, 10, and 50μM. For the first 10 experiments, concentrations of 30 and 100μM were included, but to ensure enough cells for five replicates, these concentrations were discontinued for the remaining experiments. Then, 200 μL of each concentration was added to individual 0.4‐mm thick bovine bone slices in a 96‐well plate, and left for precoating for 24 hours at 37°C in the dark.

### Bone resorption assays

Mature OCs were detached from culture flasks using accutase (Biowest BW, Nuaillé, France), and were reseeded onto the Zol‐coated bone slices at a density of 50,000 cells per well in 200‐μL media (96‐well plates). OCs were cultured for 72 hours with 25 ng/mL M‐CSF and RANKL. Subsequently, 100‐μL conditioned media was stored at −20°C for later CTx and tartrate‐resistant acid phosphatase‐ (TRACP‐) activity analyses.^(^ ^32^ ^)^ CellTiter‐Blue viability assay (Promega, Fitchburg, WI, USA) was performed in the remaining media. Bone slices were stained with toluidine blue.^(^ ^32^ ^)^ The percentage of eroded surface/bone surface (ES/BS) was analyzed by light microscopy using a 100‐point grid,^(^ ^31^, ^32^ ^)^ and all resorption cavities were subdivided into pits or trenches.^(^ ^32^, ^34^ ^)^ Pits were defined as an excavation, circular in appearance, and where the ratio between length and width of the excavation did not exceed two. Trenches were defined as an elongated and continuous excavation, and at least two times longer than its width.^(^ ^32^, ^34^ ^)^ For each donor, a dose‐response curve was fitted using a one‐phase decay curve fit to show the effect of various Zol concentrations on: (i) total ES/BS (mean *r* ^2^ for all 46 curve fits = 0.8697, median = 0.8997, range, 0.4629–0.9596), (ii) pit surface/BS (mean *r* ^2^ for all 46 curve fits = 0.7149, median = 0.7538, range, 0.2343–0.9246), and (iii) trench surface/BS (mean *r* ^2^ for all 46 curve fits = 0.8427, median = 0.8688, range, 0.3928–0.9588). Curves were used to calculate the IC50 and IC100. IC50 was defined as the halfway concentration for reaching the maximum inhibition and IC100 as the concentration for reaching maximum inhibition. Bone slices of individual experiments were blinded prior to quantification. In addition, the observer was blinded with respect to donor characteristics during the quantification of bone resorption, OC numbers, and nuclei per OC.

### CTx‐I and PINP measurements

Concentration of bone resorption marker CTx (coefficient of variation [CV] = 10%, LOD = 5 ng/mL) and formation marker PINP (CV = 10%, LOD = 0.01 ng/mL) were determined using fasting serum samples. These, as well as CTx in the conditioned media, were measured using routine chemiluminescence immunoassays, according to the manufacturer's instructions (Cobas e602 analyzer; Roche Diagnostics, Hvidovre, Denmark).

### Droplet digital RT‐PCR

Cells from each donor were lysed, RNA was extracted, and cDNA was generated.^(^ ^31^ ^)^ The copy number concentrations were measured by ddPCR using the QX100 Droplet Digital PCR system (Bio‐Rad, Hercules, CA, USA) as previously described in Møller and colleagues.^(^ ^31^ ^)^ The expression of the target genes was normalized to *GUS*. All TaqMan primer sets were used according to the supplier's instructions (Applied Biosystems, Foster City, CA, USA) as follows: GUS: Hs99999908_m1 (ViC‐MGB), cathepsin K (CatK): Hs00166156_m1 (FAM‐MGB), and farnesyl diphosphate synthase (FDPS): Hs00266635_m1 (FAM‐MGB).

### Western blot analyses

OCs from each donor were lysed and protein concentrations were determined using the Protein Bradford Protein Assay kit (protein assay dye reagent; Bio‐Rad). Then, 6‐μg protein extract was loaded on a Criterion precast 10% Bis‐Tris gel (Bio‐Rad) and was run as previously described.^(^ ^31^ ^)^ Western blotting was performed, using the antibodies: rabbit‐αhCatK pAb (Abcam, Cambridge, UK), rabbit‐αhFDPS pAb (Thermo Fisher Scientific, Waltham, MA, USA), HRP‐coupled anti‐rabbit Ab (GE HealthCare, Chicago, IL, USA), and mouse‐αhβActin mAb (Merck, Kenilworth, NJ, USA) as previously described,^(^ ^33^ ^)^ and developed using the ChemiDoc MP Imaging system (Bio‐Rad). Quantifications were performed using the Image Lab software (version 6.1.0; Bio‐Rad), and protein levels were normalized to β‐actin. Specifications of the antibodies can be found in Supplemental Table S1, and representative examples of the Western blots for both CatK and FDPS can be found in Supplemental Figure S1.

### Statistical analysis

Multiple linear regression analyses and relevant model assumptions were performed using STATA/SE, version 16 (StataCorp, College Station, TX, USA). Variance inflation factors were computed to ensure that no multicollinearity existed among predictor variables. Grubbs' test for outliers was used to exclude one outlier in the nonsmoker group (α = 0.0001). All graphs and associated statistics were performed using GraphPad Prism software, version 8 (GraphPad Software). Normal distribution was investigated using the D'Agostino & Pearson test. Correlation analyses were performed using either Spearman's rank correlation (*r*_s_) or Pearson's correlation (*r* ^2^). Paired comparisons between groups were performed using the Wilcoxon signed rank test. All figures were made using CorelDRAW X5 (Corel Corporation).

## Results

In the current study, 46 healthy female blood donors, between 40 to 66 years of age, were included. The described population is a subset of the donor population we have previously reported on.^(^ ^31^ ^)^ In the previous study, we reported on how donor variations affected the resorptive activity (control condition) of OCs generated in vitro. Here we report on how donor variations affect the sensitivity of OCs to Zol treatment in vitro.

### Zol is not equally potent on OCs generated from different individuals

**Fig 1:**
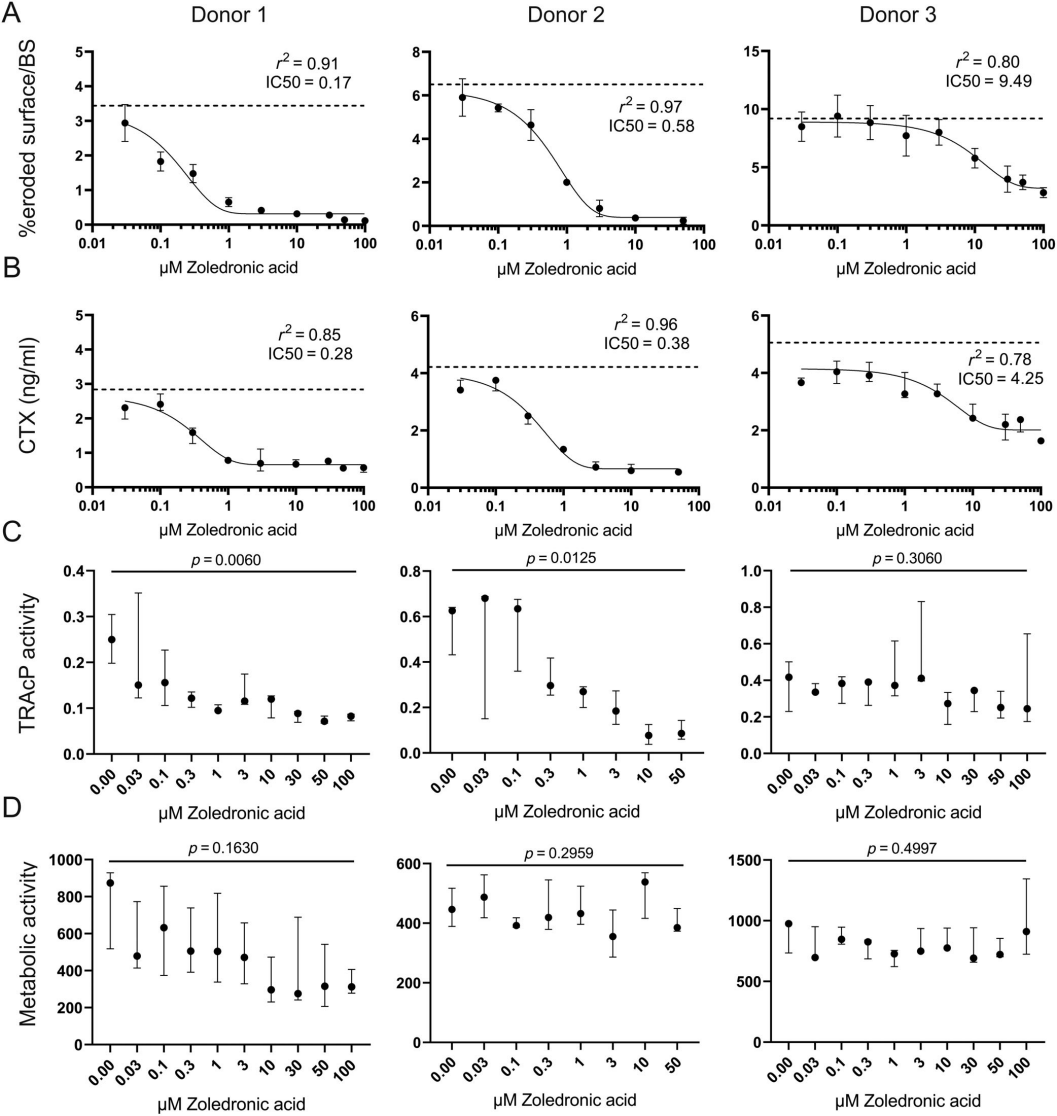
Examples of responses to Zol for OCs generated from three different donors. Responses to Zol were determined by: (*A*) The IC50 value, based on total eroded bone surface after 3 days of resorption (*n* = 5). Each data point represents the mean ± SD; baseline is indicated by a dotted line. (*B*) The IC50 value, based on the concentration of CTx in the conditioned media, after 3 days of resorption (*n* = 3). Each data point represents the median ± range; baseline is indicated by the dotted line. (*C*) Tartrate‐resistant acid phosphatase (TRACP) activity (arbitrary units; *n* = 3); statistics: Kruskal‐Wallis test. Each data point represents the median + range. (*D*) Metabolic activity (arbitrary units; *n* = 3) statistics: Kruskal‐Wallis test. Each data point represents the median and range.

Figure 1 shows representative examples of the OC activity following Zol exposure for three donors. From the ES/BS (Fig. 1*A*) and CTx levels in conditioned media (Fig. 1*B*), a dose‐response curve was fitted using a one‐phase decay curve fit. From these curves, the IC50 values were calculated for each donor. The IC50 is used here as a measure of the osteoclastic sensitivity to Zol. Fig. 1*A* shows a comparison of the extent of eroded bone surface at different concentrations of Zol for three donors. The dotted line indicates the extent of bone resorption at baseline, which for these three donors varied from 3.5% to 9%. Visually, it is clear that Zol decreases the bone‐resorptive activity for all the donors. However, the OCs of donor 1 appear to decrease their resorptive activity at quite low concentrations of Zol (IC50: 0.17μM Zol), whereas those from donor 2 are more tolerant (IC50: 0.58μM Zol), and those of donor 3 show an even greater tolerance (IC50: 9.49μM Zol). It is also worth noticing that the ES/BS was reduced to <1% for donors 1 and 2, whereas it reached a plateau at around 4% for donor 3. Zol treatment has both a lower potency and a lower effect on the OCs generated from donor 3 compared with those from donors 1 and 2.

As another measure for bone‐resorption activity, CTx in the conditioned media of the cultures was used. OCs of donor 1 were most sensitive to Zol (IC50: 0.28μM Zol), OCs of donor 2 were less sensitive (IC50: 0.38μM Zol), whereas OCs from donor 3 were the least sensitive (IC50: 4.25μM Zol). Fig. 1*C* shows the corresponding TRACP‐activity. For donors 1 and 2, increasing Zol concentrations resulted in a significant decrease in TRACP activity (*p* = 0.0060 and *p* = 0.0125, respectively), whereas for donor 3, no significant decrease in TRACP‐activity was found (*p* = 0.3080). There was no significant decrease in metabolic activity for any of the donors (Fig. 1*D*; *p* = 0.1630, *p* = 0.2959, *p* = 0.4997).

### There is a 210‐fold difference in sensitivity to Zol of OCs generated from different donors

**Fig 2:**
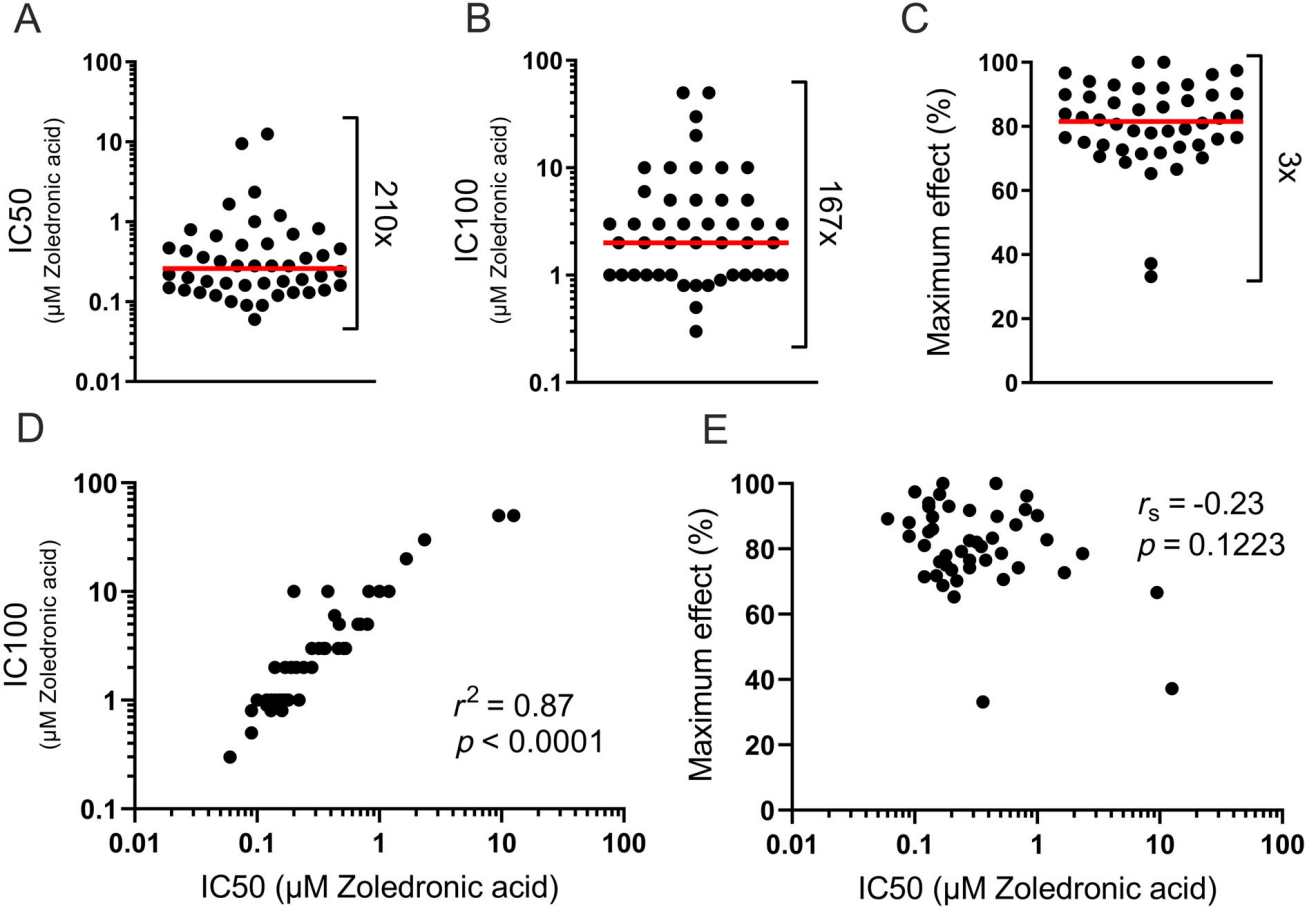
The sensitivity to Zol varies significantly among OC preparations (based on total eroded surface after 3 days of resorption). (*A*) Variation in IC50. (*B*) Variation in IC100. (*C*) Variation in maximum effect of Zol. How much (in percent) Zol could reduce baseline resorption levels. (*D*) Correlation between IC50 and IC100. (*E*) Correlation between IC50 and maximum effect. Statistical correlation analyses were performed using either Pearson's correlation (*r* ^2^) or Spearman's rank correlation (*r*_s_). Each data point represents the results obtained from osteoclasts generated from an individual donor (*n* = 46); the red line represents the median.

Figure 2*A* illustrates the variation in IC50 when using the OCs generated in vitro from the 46 female donors. IC50 values varied from 0.06 to 12.57μM Zol among the OC preparations with a median of 0.26μM. Thus, there is a remarkable 210‐fold difference in the sensitivity to Zol. With respect to IC100, a 167‐fold variation was observed (Fig. 2*B*). IC100 varied from 0.3μM to 50μM, with a median of 2μM Zol. Moreover, it was assessed how much (in percent) Zol could reduce the baseline resorption level (maximum effect). The median of the maximum effect was 82% ranging from 33% to 100% (Fig. 2*C*). The IC50 value was found to strongly correlate with IC100 (*r* ^2^ = 0.87, *p* < 0.0001; Fig. 2*D*), which could be expected because they are both measures of potency. However, when comparing IC50 with the maximum effect of treatment, no correlation was found (Fig. 2*E*).

### Metabolic activity of OCs in vitro decreases with Zol treatment, but not for all donors

**Fig 3:**
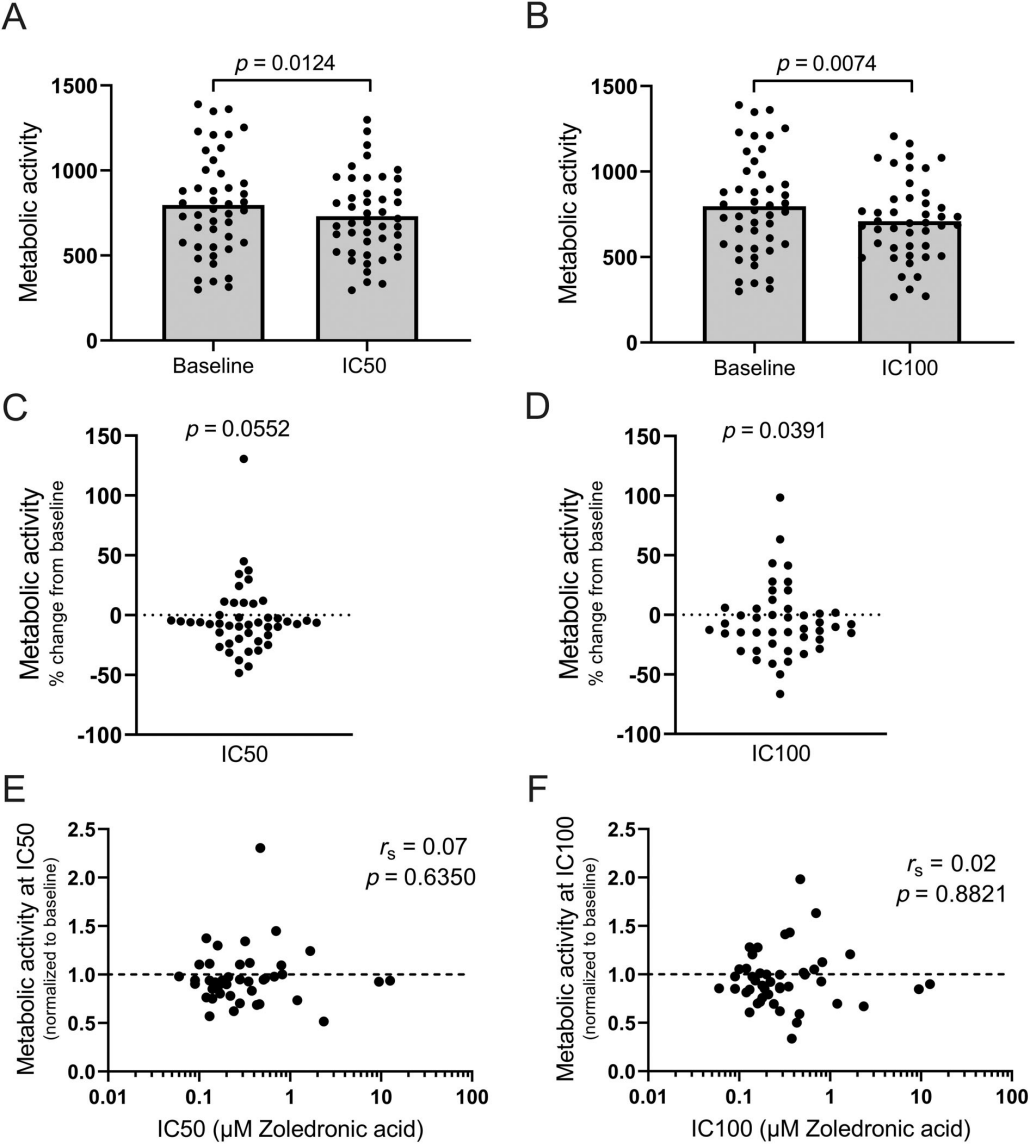
Zol treatment decreases the metabolic activity of OCs in vitro, but not for all donors. (*A*) Metabolic activity of OCs at baseline compared to the metabolic activity of OCs at IC50 (based on total eroded surface). (*B*) Metabolic activity of OCs at baseline compared to the metabolic activity of OCs at IC100. (*C*) The percent change in metabolic activity from baseline to IC50 (based on total eroded surface). (*D*) The percent change in metabolic activity from baseline to IC100. **(** *E*) Comparison of the IC50 value with the metabolic activity at IC50 (normalized to baseline; based on total eroded surface). (F) Comparison of the IC50 value with the metabolic activity at IC100 (normalized to baseline). Baseline is indicated by the dotted line. Statistical tests: (*A*) Wilcoxon matched‐pairs signed rank test, (*B*) paired *t* test, (*C* + *D*) One sample Wilcoxon test, and (*E* + *F*) Spearman's rank correlation (*r*_s_). For all graphs, each data point represents the results obtained from OCs generated from an individual donor (*n* = 46).

Comparing metabolic activity of the in vitro‐generated OCs at baseline and at IC50 shows that there was, on average, a significant decrease in metabolic activity at IC50 (*p* = 0.0124; Fig. 3*A*). A similar observation was made for IC100 (*p* = 0.0074; Fig. 3*B*). However, the average metabolic activity only decreased by 8% (Fig. 3*A*) and 11% (Fig. 3*B*), and a substantial variation was found at both IC50 (Fig. 3*C*) and IC100 (Fig. 3*D*). In fact, for some of the OC preparations, an increased metabolic activity was observed in response to Zol. For 24%, the metabolic activity increased at the IC50 compared with baseline (Fig. 3*C*), whereas it increased for 30% at IC100 compared with baseline (Fig. 3*D*). Finally, the IC50 or IC100 value for each OC preparation was matched with the level of metabolic activity (Fig. 3*E*,*F*, respectively). No correlations were found.

### OC preparations with high bone resorptive activity and many pit cavities are less sensitive to Zol

**Fig 4:**
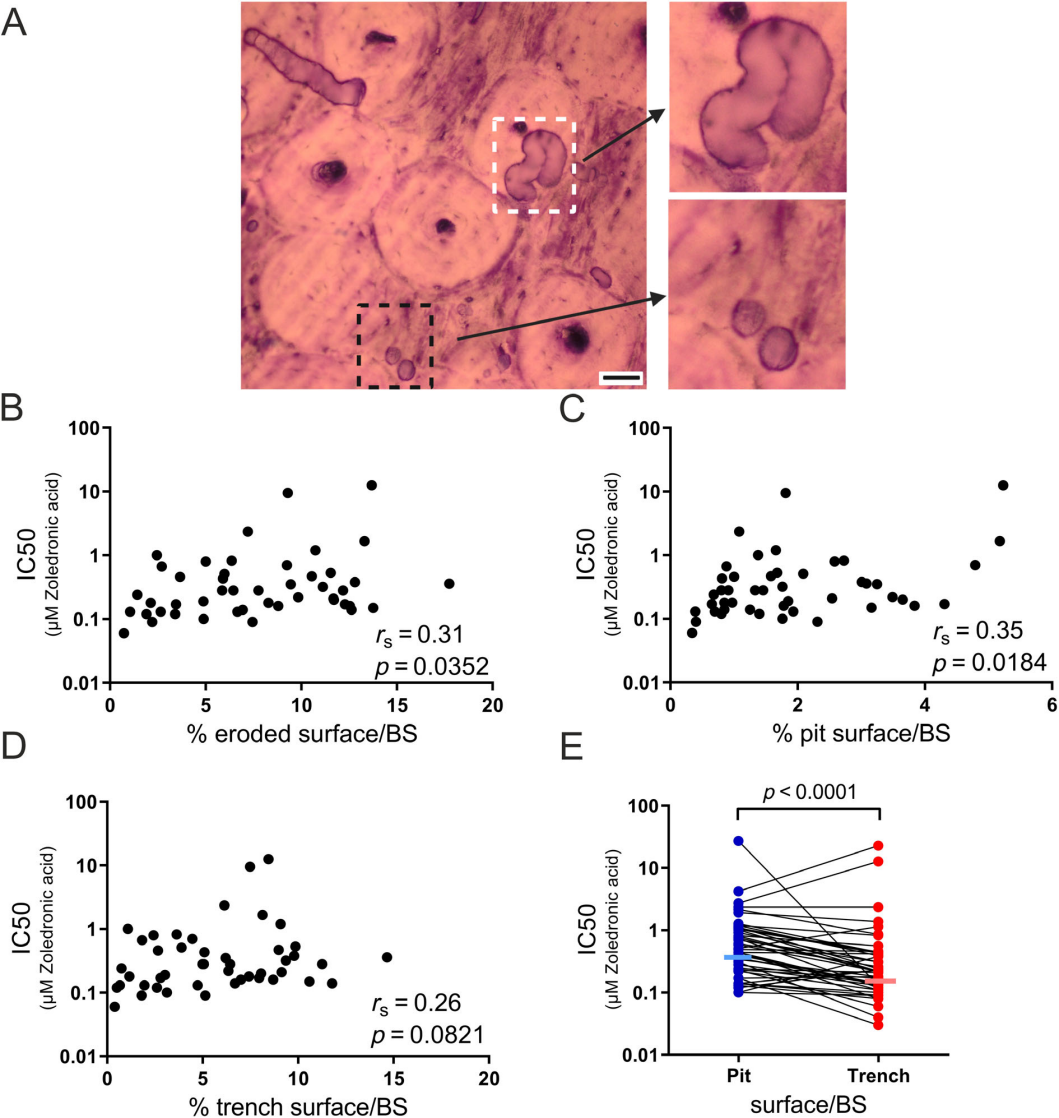
OC activity and resorption mode (pits and trenches) affect the sensitivity to Zol. (*A*) Examples of pits (stipulated black box) and trenches (stipulated white box) on a cortical bovine bone slice stained with toluidine blue. Enlargement of these can be seen on the right. The black scale bar corresponds to 50 μm. (*B*) Correlation analysis between the total eroded surface at baseline and the IC50 value (based on total eroded surface) for each OC preparation. (*C*) Correlation analysis between the extent of pit surface/bone surface (BS) at baseline and the IC50 value (based on total eroded surface) for each OC preparation. (*D*) Correlation analysis between the amount of trench surface/BS at baseline and the IC50 value (based on total eroded surface) for each OC preparation. (*E*) Matching the IC50 value for pit and trench surfaces in the same culture (medians are indicated by horizontal lines). Statistical correlation analyses were performed using (*B*–*D*) Spearman's rank correlation (*r*_s_) and (*E*) Wilcoxon matched‐pairs signed rank test. Each data point represents the results obtained from OCs generated from an individual donor (*n* = 46).

We have previously shown a >20‐fold difference in the amount of bone resorption among OC preparations from different donors.^(^ ^31^ ^)^ Therefore, it was relevant to investigate if this variation could be of importance for the sensitivity to Zol. Examples of resorption cavities analyzed (pits and trenches) can be seen in Fig. 4*A*. Figure 4*B* shows a positive correlation between the total ES/BS and the corresponding IC50 value (*p* = 0.0352). When focusing on the two different types of resorption contributing to the total ES/BS, we found that pit surface/BS at baseline correlated significantly with the IC50 value (*p* = 0.0184; Fig. 4*C*) while this only reached near‐significance for trench surface/BS at baseline (*p* = 0.0821; Fig. 4*D*).

### Trench‐forming OCs are more sensitive to Zol in vitro than pit‐forming OCs

Rather than comparing the resorption modes for the relation between resorption activity level and IC50 in every culture, we compared the IC50s of pit and trench ESs within the same culture. The test clearly shows that OCs making trenches in general are more sensitive to Zol than pit‐forming OCs in the same culture (*p* < 0.0001; Fig. 4*E*). The overall medians for pits and trenches were 0.47 and 0.22μM Zol, respectively.

### In vitro‐measured OC sensitivity to Zol correlates with donors' smoking habits and the average number of nuclei per OC in vitro

To investigate what could explain the observed 210‐fold variation in sensitivity to Zol in vitro among donors, we performed a multiple linear regression analysis combining in vitro and in vivo collected data (Table 2). The dependent variable, the IC50 value determined for the ES/BS, was log‐transformed prior to running the analysis. The test showed that the smoking habits and the average number of nuclei per OC in vitro were the best predictors of the observed variation in sensitivity to Zol among OC preparations. Altogether, this model explained 30% of the observed variation. Smoking status was positively correlated with the IC50 value (*p* = 0.010), demonstrating that OCs generated from smokers are less sensitive to Zol than OCs from nonsmokers. The average number of nuclei per OC was also positively correlated with the IC50 value (*p* = 0.029), showing that the more nuclei an OC on average has, the less sensitive it is to Zol (Table 2).

**Table 2:** Bone Resorptive Activity of OCs in vitro Correlates With the Smoking Status and the Number of Nuclei per OC

Dependent variable	*R* ^2^	Independent variable	Coefficient	SE	*t*	*P* Value
IC50_log^a^	0.30	Age (years)	−0.001	0.03	−0.00	0.997
		Menopause (0 = post‐, 1 = pre‐)	0.613	0.46	1.34	0.190
		Height (m)	−2.213	3.10	−0.71	0.480
		Weight (kg)	−0.002	0.01	−0.15	0.880
		**Smoking (0 = no, 1 = yes)**	**1.289**	**0.47**	**2.73**	0.010*
		Comorbidity (0 = no, 1 = yes)	−0.118	0.42	−0.28	0.779
		CTx in vivo (ng/mL)	−0.964	1.52	−0.63	0.530
		PINP in vivo (ng/mL)	0.022	0.02	1.39	0.174
		#OCs	0.002	0.01	0.41	0.686
		**#nuclei/OC**	**0.314**	**0.14**	**2.28**	0.029*
		_cons	0.375	5.32	0.07	0.944

^a^ Based on total eroded bone surface after 3 days of resorption.

### Sensitivity to Zol correlates with the amount of mature CatK protein in the cell, but not the amount of FDPS

**Fig 5:**
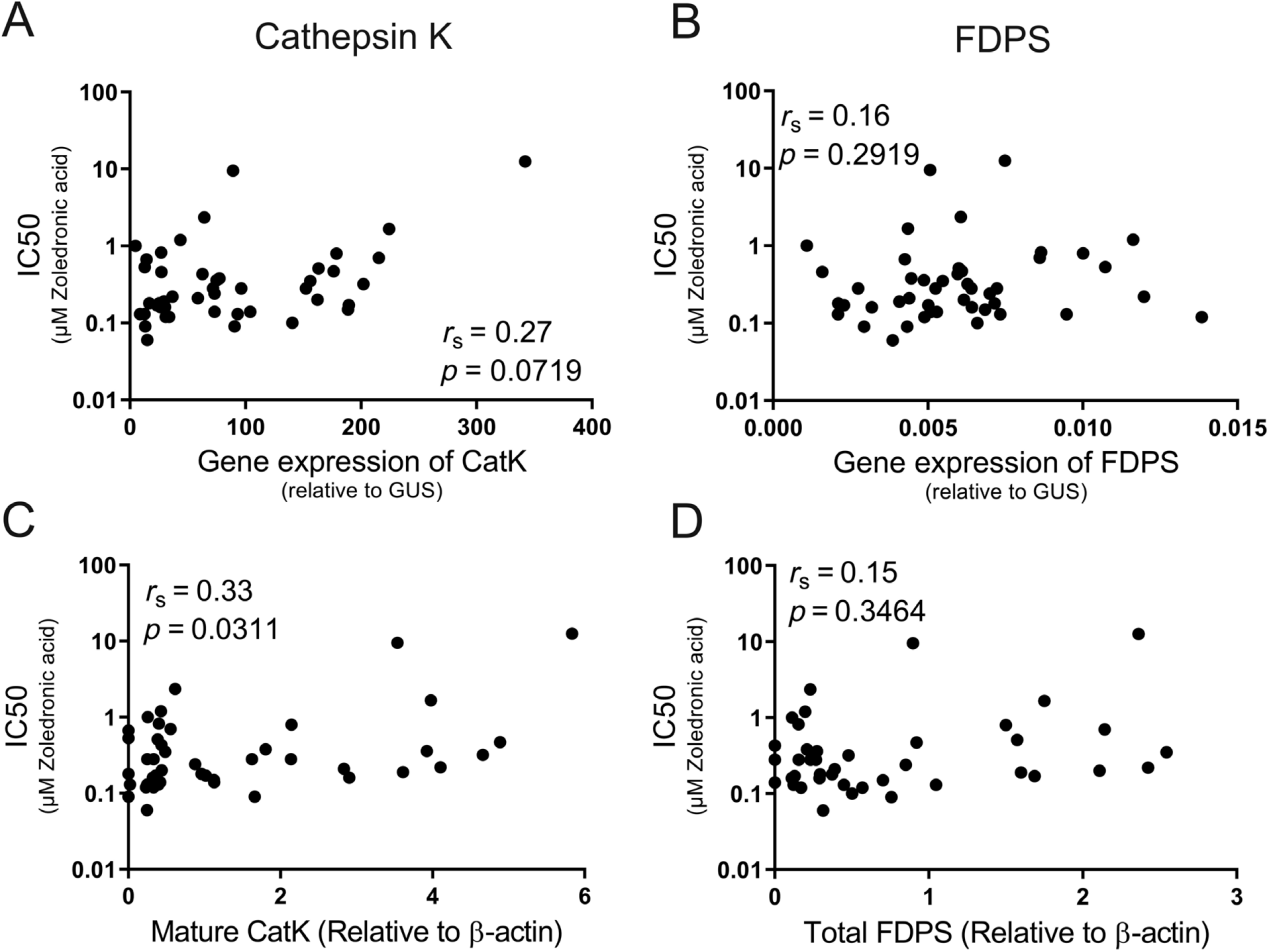
The sensitivity to Zol correlates with the amount of mature CatK in the cell, but not the amount of FDPS. Correlation analyses between the IC50 value (based on total eroded bone surface) for each OC preparation and: (*A*) *CTSK* gene expression, (*B*) *FDPS* gene expression, (*C*) the amount of mature CatK protein, and (*D*) the total amount of FDPS protein. Statistical correlation analyses were performed using Spearman's rank correlation (*r*_s_). Each data point represents the results obtained from OCs generated from an individual donor (*n* = 45).

Because the extent of eroded bone surface at baseline was shown to affect the IC50 of Zol, and protein levels of active CatK are a strong determinant for the eroded bone surface at baseline,^(^ ^31^ ^)^ we investigated whether CatK could be a determinant for sensitivity to Zol. The gene expression of *CTSK* showed a trend toward a positive correlation with the IC50 value (*p* = 0.0719; Fig. 5 *A*). However, the protein‐level of mature CatK (determined by Western blotting; Supplemental Figure S1), showed a positive correlation with the corresponding IC50 value (*p* = 0.0311), demonstrating that those OC preparations that were the least sensitive to Zol had a higher protein level of mature CatK (Fig. 5*C*). Moreover, the gene expression (*p* < 0.0001) and protein levels (*p* < 0.0001) of CatK were found to correlate with the average number of nuclei per OC (Supplemental Fig. S2*A*,*B*). No difference was found in the average number of nuclei per OC from OC preparations of smokers and nonsmokers (data not shown). A comparison of the gene expression of *FDPS* with the IC50 value for each donor showed that an increased gene expression of *FDPS* did not seem to alter the OCs' sensitivity to Zol (Fig. 5*B*). Correspondingly, no correlation was found between the total amount of FDPS protein and the IC50 value (Fig. 5*C*; as determined by Western blotting; Supplemental Figure S1). Finally, the gene expression (*p* = 0.1832) and protein levels (*p* = 0.2668) of FDPS were not found to correlate with the average number of nuclei per OC (Supplemental Fig. S2*C*,*D*).

## Discussion

We confirmed our hypothesis that human OCs generated from different individuals show a variable sensitivity to Zol in vitro and that intrinsic differences between individuals can explain this variation. We found a more than 200‐fold difference in sensitivity (IC50) to Zol among OCs generated from different individuals, as well as a large variation in IC100. As expected, there was a clear correlation between IC50 and IC100. These are both good measures for comparing the potency of Zol between OC preparations; however, for a more comprehensive description of the effect of Zol, the drug's maximum effect (% inhibition from baseline) was also compared. A threefold difference in the maximum effect was found, demonstrating that for some donors the highest concentration of Zol could inhibit 100% of the resorption, whereas for others only 33% of the resorption. However, it is important to note that three‐quarters of the donors reached more than 75% inhibition. Of note, the OC preparations that show a high sensitivity to Zol (low IC50) were not necessarily the ones that gained the greatest effect on reducing bone resorption in vitro. A likely cause for this may be that the maximum effect of Zol is even more sensitive to the overall resorption level (data not shown) than IC50. This creates some noise that may explain why correlation between these two parameters only reached marginal significance (*p* = 0.1223).

When considering the potential clinical relevance of this in vitro study, it is of major interest to determine if the used concentrations of Zol are clinically relevant and comparable to the in vivo concentrations obtained in patients treated with Zol. Patients with cancer treated for bone disease receive 4 mg (every 3 to 4 weeks), whereas patients with osteoporosis receive 5 mg (per year) as an intravenous infusion.^(^ ^35^, ^36^ ^)^ Immediately after infusion of 4‐mg Zol, a plasma concentration of around 2.4μM is reached,^(^ ^37^ ^)^ and 24 hours after administration of Zol, around half of the dose is excreted into the urine.^(^ ^37^, ^38^ ^)^ Thus, approximately half of the Zol administered will be retained on accessible bone surfaces. However, plasma concentrations are not an exact measure of how much Zol has truly bound to a given bone surface within the human body. The surface area of the bone varies among individuals, simply because of individual differences in height and weight, for example. Moreover, studies have suggested that bisphosphonates are not evenly distributed throughout the skeleton,^(^ ^39^ ^)^ but that uptake is highest in cancellous bone and the axial skeleton, and less so in the appendicular bones and the head.^(^ ^39^ ^)^ However, with concentrations ranging between 0.03 and 50μM Zol, our experimental design ensures that our Zol doses are approximately within the biological range.

With a 210‐fold difference in OC sensitivity to Zol, our data support the hypothesis that OCs from different individuals respond differently to Zol. Our findings are supported by in vivo studies on patients with breast cancer, which show that a substantial fraction of patients on long‐term antiresorptive treatment still develop SREs,.^5^, ^6^ ^)^ In addition, a considerable minority of patients with osteoporosis have a poor response to treatment.^(^ ^8^ ^)^ In fact, our study found that the current Zol dosing may not be sufficient to efficiently reduce bone resorption for certain patients, whereas for others it may be more than necessary. This is important when considering the potential consequences of prolonged bisphosphonate use, such as atypical femoral fractures, atrial fibrillation, and osteonecrosis of the jaw.^(^ ^40^ ^)^ To optimize treatment with Zol, a more personalized approach is desirable: Our study is a step in this direction.

The variation in IC50 was found to correlate with both in vitro and in vivo characteristics of the donors. OCs from smokers were found to be less sensitive to Zol treatment than those from nonsmokers. Smoking has previously been identified as a risk factor for osteoporosis,^(^ ^41^ ^)^ and has been found to cause an imbalance in bone turnover, leading to lower BMD, consequently rendering bone increasingly more prone to osteoporosis and fractures.^(^ ^42^, ^43^, ^44^, ^45^ ^)^ Smoking has also been shown to increase breast cancer risk^(^ ^46^ ^)^; breast cancer cells exposed to cigarette smoke extract display an increased motility and cell adhesion in animal studies.^(^ ^47^, ^48^ ^)^ Cairoli et al^(^ ^49^ ^)^ have shown that current smoking is associated with a poor response to alendronate or risedronate in postmenopausal women with primary osteoporosis. Our data support the findings of Cairoli and colleagues,^(^ ^49^ ^)^ and provide a possible mechanistic explanation for the correlation between current smoking and inadequate response to bisphosphonate treatment. However, it is important to note that our study was not designed or powered to address the effects of smoking; hence, we had a small number of smokers in our study. Nevertheless, our findings are of potential clinical relevance. Therefore, we have recently initiated a new trial to address if smoking affects the potency of Zol on OCs both in vivo and in vitro.

Multiple linear regression analyses also showed that the average number of nuclei per OC correlated significantly with the sensitivity to Zol in vitro. Surprisingly, this was independent of the number of multinucleated OCs present in the culture. The correlation between the number of nuclei per OC and sensitivity to Zol is an interesting observation as the size of OCs (number of nuclei per OC) in near proximity to bone metastases have been reported to be abnormally large with respect to number of nuclei in both humans^(^ ^50^ ^)^ and mice.^(^ ^51^ ^)^ Thus, an increase in the nucleation of OCs alone might be a contributing factor to the incomplete suppression of bone resorption by bisphosphonates in some patients with cancer and bone metastases.^(^ ^5^, ^6^, ^7^ ^)^ Of note, hypernucleated OCs can be observed in bone biopsies of patients with osteoporosis treated with long‐term oral alendronate.^(^ ^52^ ^)^ They are generally assumed to be inactive; however, whether these giant cells have any function or if they alter the sensitivity to bisphosphonates remains an interesting question. Donors with OCs that are more actively resorbing bone at baseline were found to have OCs that were less sensitive to Zol. Previously, it has been shown that the amount of bone resorption (at baseline) correlates with the number of nuclei per OC.^(^ ^31^, ^53^ ^)^ Therefore, the correlation between in vitro bone resorption and the OCs' sensitivity to Zol is likely to be a direct consequence of the increased number of nuclei per OC. The quantification of OCs and nuclei per OC was based on analyses of the OCs as they were in the culture flasks before they were reseeded on bone slices. They therefore reflect the precursors' ability to differentiate into mature multinucleated OCs and the average nucleation status of the OCs that were reseeded onto bone slices. Preferably, these quantifications should have been performed on the cells after they were reseeded onto the bone slices. Unfortunately, this was not possible.

We have previously shown that the type of resorption cavities (pit and trench formation) varies with both age^(^ ^31^ ^)^ and gender.^(^ ^34^ ^)^ Therefore we speculated if OC sensitivity to Zol was associated with the type of OCs that makes pits or trenches. Therefore, bone‐resorption cavities were subdivided into pits and trenches,^(^ ^34^, ^54^ ^)^ and the IC50 value for both pit‐ and trench‐related bone resorption was assessed. When doing so, we found that pit‐forming OCs were less sensitive to Zol than trench‐forming OCs. In support of this, OC preparations generating a high pit surface were less sensitive to Zol, whereas no correlation was found between trench surface and sensitivity to Zol. Comparable observations have been reported for alendronate.^(^ ^55^ ^)^ The reason for these differences in sensitivity between the resorption types is unclear, but a possible reason may be that OCs making trenches come in contact with more bone surface during bone resorption and may therefore take‐up more Zol than those making pits. In addition, trench‐forming OCs are more demanding regarding function of small GTPase‐dependent pathways, such as migration, vesicular transport, cytoskeletal rearrangements, etc.^(^ ^33^, ^34^ ^)^ However, more research is needed to understand the potential impact of these findings. Such investigations could be of importance considering the impact of age and gender^(^ ^31^, ^34^ ^)^ on the resorptive behavior of OCs (pits or trenches).

One of the most important enzymes in osteoclastic resorption is the collagenolytic enzyme CatK.^(^ ^56^ ^)^ Therefore, changes in gene expression levels and the corresponding amount of CatK protein were investigated for all donors and were compared with their OCs' sensitivity to Zol. We found that the protein levels of mature CatK correlated positively with the IC50 value. As a correlation was found between the number of nuclei per OC and both the gene expression and proteins levels of CatK, one could speculate that OCs with a high number of nuclei have more “power” to produce CatK, and as a result are more resilient to the effects of Zol on their resorptive activity. However, this may seem contradictory to our finding that OCs making trenches are most sensitive to Zol, especially because we have previously published that OCs making trenches have more active CatK.^(^ ^57^, ^58^ ^)^ We have shown that OCs making trenches do so by first making a pit and can only shift to the trench mode when reaching a certain resorption depth.^(^ ^59^ ^)^ In the presence of Zol, there is less chance to reach this stage because the OC becomes progressively inhibited by Zol. And if it reaches this stage, there is less chance that it can go on resorbing for a long time because it internalizes more and more Zol. We believe that this may explain why OCs making trenches are more sensitive to Zol than those making pits. Still, the more CatK, the better chances are to reach the trench mode (and even to go on); therefore, more Zol is needed to antagonize resorption. This is most likely why the IC50 increases with increasing CatK.

Surprisingly, neither the gene expression nor protein levels of FDPS had an effect on the OC sensitivity to Zol in our study. A recent study reported that single nucleotide polymorphisms in the FDPS gene correlate with the effect of bisphosphonates in women with postmenopausal osteoporosis.^(^ ^60^ ^)^ Therefore, we were surprised that the FDPS levels had no detectable effect on OCs' sensitivity to Zol. A possible explanation could be that FDPS activity is more relevant for Zol sensitivity rather than gene expression levels. Assays have been developed to do such an analysis,^(^ ^20^ ^)^ but unfortunately, this type of assay would not have been feasible using our current experimental design. In this regard, it is worth noting that a SNP (rs2297480) in the gene‐encoding FDPS has been found to affect the in vivo sensitivity to aminobisphosphonates, but only when the individuals were homozygotes for the minor allele.^(^ ^61^ ^)^ It would therefore be interesting to evaluate whether this SNP could explain some of the variation we have observed; however, this would demand a larger sample size than available in our current study. In general, further studies are needed to understand the implications of our findings with respect to both CatK and FDPS.

Zol treatment has previously been reported to induce apoptosis in OCs.^(^ ^62^ ^)^ The mechanism by which apoptosis is induced is believed to be through the inhibition of FDPS.^(^ ^14^, ^63^ ^)^ In our study, mature OCs were exposed to different concentrations of Zol for 72 hours; this exposure did indeed reduce the metabolic activity. However, it did not induce widespread cell death, even at the highest concentrations of Zol (50 or 100μM Zol) within the 72 hours. In fact, for 30% of the donors the metabolic activity surprisingly increased following Zol treatment. It is important to remember that metabolic activity is not necessarily the same as cell viability.^(^ ^64^ ^)^ It is possible that the treatment changes the metabolic activity without affecting cell number or viability. Another possibility is that Zol inactivates OCs within 72 hours, and that apoptosis is only visible as a secondary phenomenon. One could speculate that the donors with a low OC sensitivity to Zol were also those with an unchanged or increased metabolic activity; however, this was not the case (Fig. 3*E*,*F*), indicating that it is not necessarily a Zol‐induced decrease in OC metabolism that is responsible for the observed variation in sensitivity to Zol among OCs from different individuals.

In summary, our data revealed a more than 200‐fold difference in the sensitivity to Zol, using in vitro‐generated OCs from healthy donors. Through multiple linear regression analyses combining in vitro and in vivo data, we found that in vitro‐differentiated OCs from smokers are less sensitive to Zol than those from nonsmokers, and that OCs with a higher number of nuclei per OC are less sensitive to Zol. The variation in OC sensitivity to Zol correlated with the amount of mature CatK protein for each OC preparation. Surprisingly, neither the gene expression nor protein levels of FDPS could explain the observed variation in sensitivity to Zol.

There are without a doubt many contributing factors to the effectiveness of Zol, both for patients with cancer and osteoporosis. However, from the presented results we speculate that individual differences at the level of the OC contribute to the observed variations among patients, unrelated to the given disease, bisphosphonate retention, bone turnover levels, and kidney clearance. Our data suggest why such substantial variation in the effectiveness of treatment is observed among patients receiving bisphosphonates, and highlight the need for personalized approaches in the treatment of both cancer‐induced bone disease and osteoporosis.

## Disclosures

The authors declare no competing interests.

## AUTHOR CONTRIBUTIONS

**Anaïs Møller:** Data curation; formal analysis; investigation; methodology; funding acquisition; project administration; validation; writing‐original draft; writing‐review and editing. **Jean‐Marie Delaissé:** Conceptualization; funding acquisition; methodology; resources; supervision; writing‐review and editing. **Jacob Olesen:** Data curation; investigation; methodology; writing‐review and editing. **Troels Bechmann:** Conceptualization; methodology; resources; writing‐review and editing. **Jonna Madsen:** Conceptualization; data curation; methodology; writing‐review and editing. **Kent Søe:** Conceptualization; formal analysis; funding acquisition; methodology; project administration; resources; supervision; validation; writing‐original draft; writing‐review and editing.

## Data accessibility statement

The data sets generated and/or analyzed during the current study are available from the corresponding author upon reasonable request.

### Peer Review

The peer review history for this article is available at https://publons.com/publon/10.1002/jbm4.10412.

## Supporting information


**Appendix S1**. Supplementary Information
