# Supplementary material for: Zoledronic Acid Is Not Equally Potent on Osteoclasts Generated From Different Individuals
Source: JBMR Plus. 2020 Sep 29;4(11):e10412. doi: 10.1002/jbm4.10412 (PMC7657394; doi:10.1002/jbm4.10412)
Supplement: Supplementary file 1 — Appendix S1. Supplementary Information [file JBM4-4-e10412-s001.docx]

**Supplemental Material**

**Supplemental Table 1** Description of antibodies for Western blotting.

| **Product name** | **Source and**  **Catalog #** | **Host**  **species** | **Application** | **Dilution** | **Application**  **Specific details** |
| --- | --- | --- | --- | --- | --- |
| Anti-Cathepsin K antibody - Catalytic domain | Abcam  ab49893 | Rabbit | WB | 0.26µg/ml | 3% BSA in TBS-  Tween (0.1%)  Overnight 4°C |
| FDPS Polyclonal Antibody | Thermo Fisher Scientific  PA5-28228 | Rabbit | WB | 0.94µg/ml | 3% BSA in TBS-  Tween (0.1%)  Overnight 4°C |
| Monoclonal Anti-β-Actin antibody | Merck  A2228 | Mouse | WB | 0.80µg/ml | 3% BSA in TBS-  Tween (0.1%)  30min RT |
| Amersham ECL Western Blotting Detection System Anti-mouse HRP conjugate and Anti-rabbit HRP conjugate | GE Healthcare  RPN2108 | - | WB | 1:5000 | Used according to instructions by the supplier |


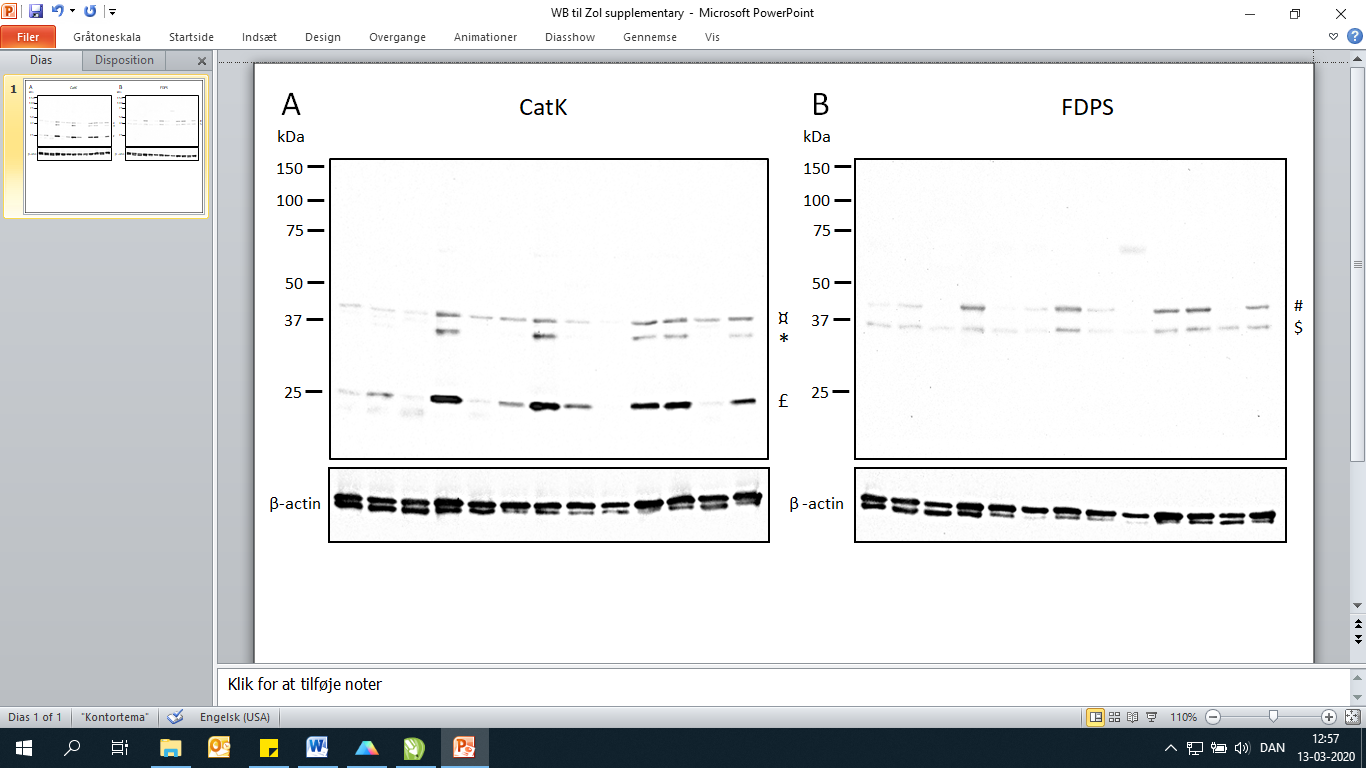


Supplemental Figure 1 A) Example of a Western blot of CatK protein in OC preparations for 13 different donors. ¤, pre-pro-CatK; *, pro-CatK; £, mature CatK. B) Westerns blot of FDPS protein in OC preparations (same cell lysates as in A). $, band 1; #, band 2. (The corresponding unedited Western blots can be found in Supplemental Figure 3).


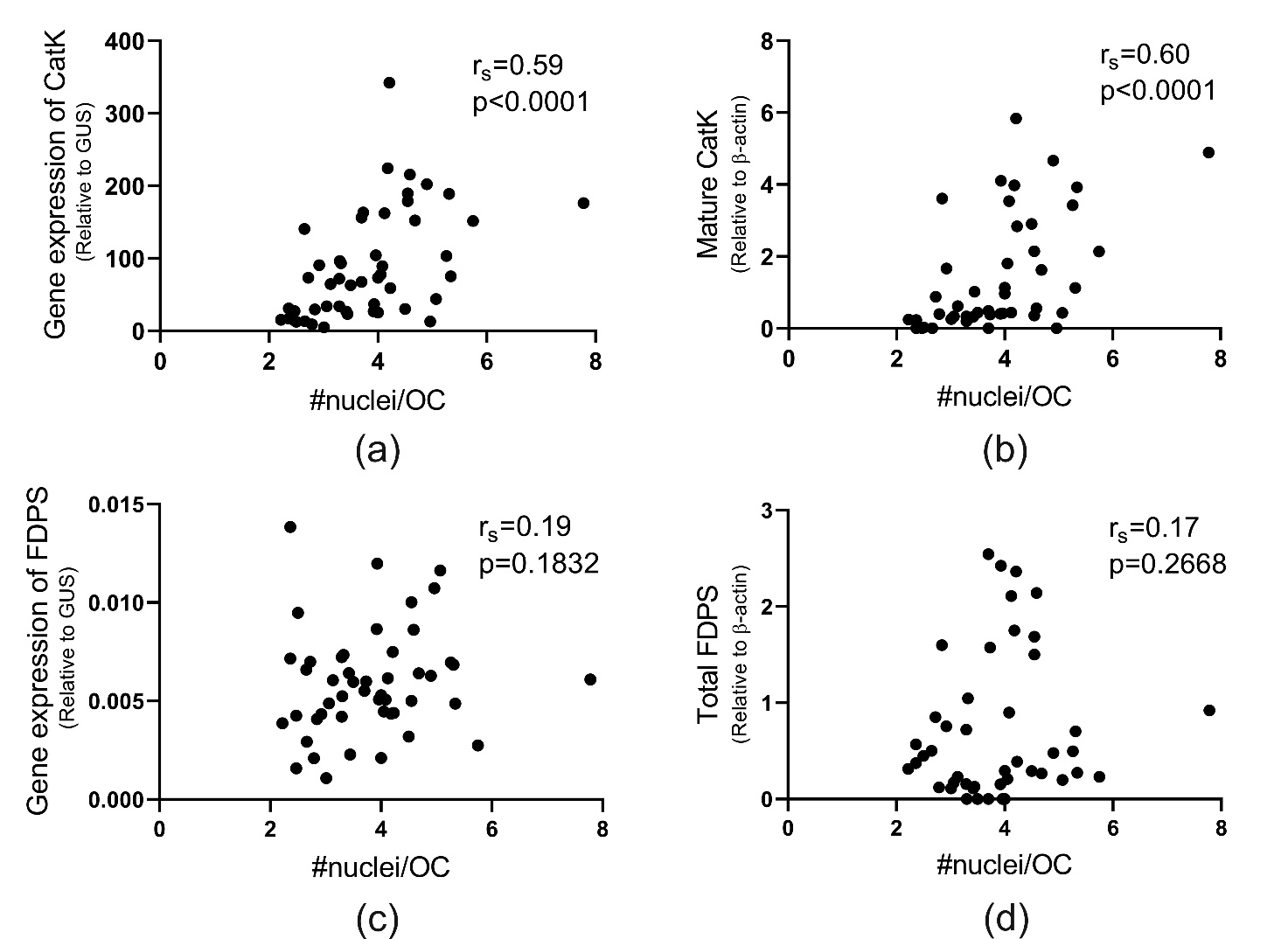


Supplemental Figure 2 Gene expression and protein levels of CatK increase with the number of nuclei per OC, while FDPS does not. Comparison of the number of nuclei per OC with: (a) the gene expression of CatK, (b) the protein levels of mature CatK, (c) the gene expression of FDPS, and (d) the protein levels of FDPS. Statistical correlation analyses were performed using either Pearson's correlation (r^2^) or Spearman's rank correlation (r_s_). Each dot represents the results obtained from OCs generated from an individual donor (n=45).


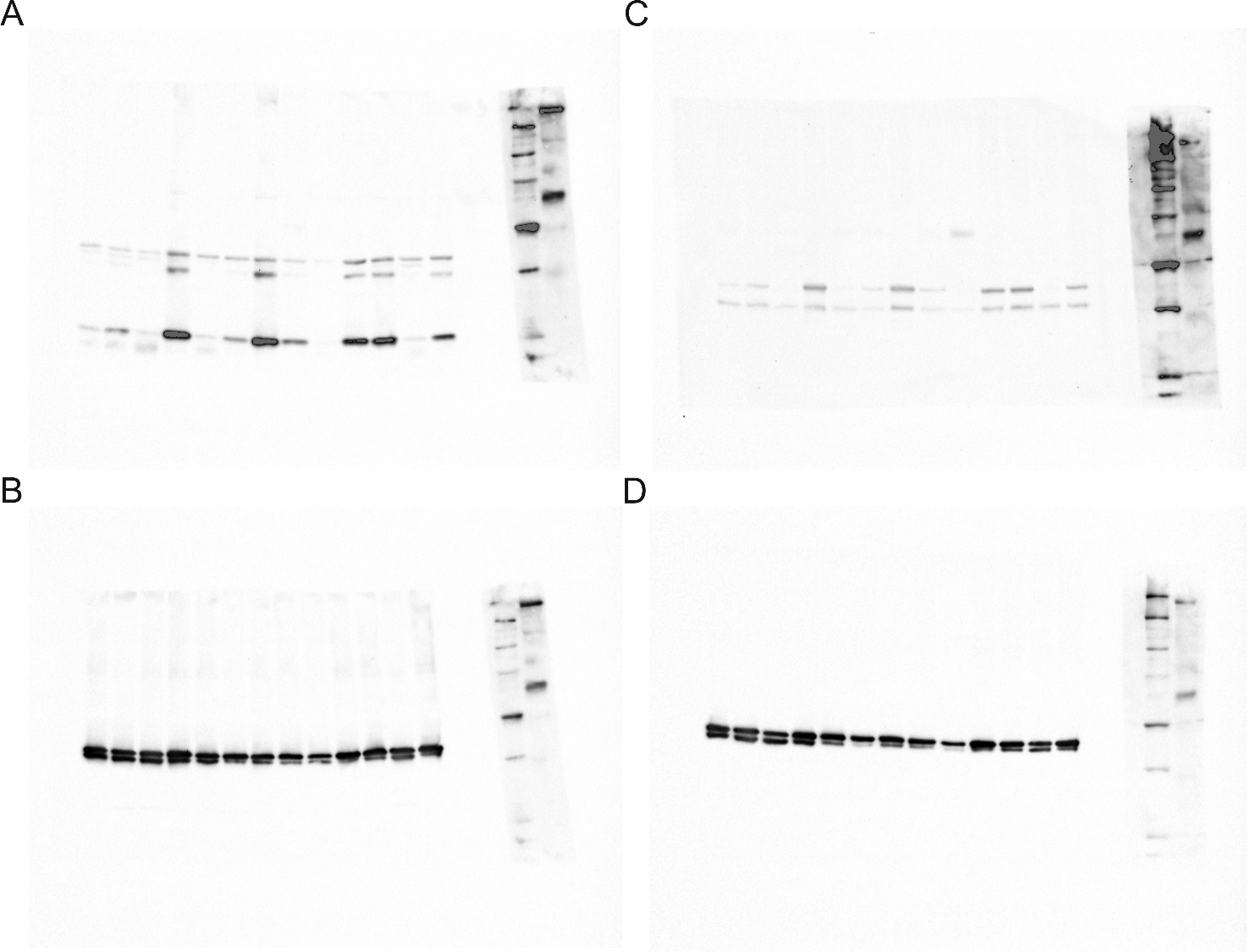


Supplemental Figure 3 The unedited Western blots shown in Supplemental Figure 1. A) Western blot of CatK protein in OC preparations for 13 different donors. B) Corresponding Western blot of β-actin. C) Western blot of FDPS protein in OC preparations for the same 13 donors. D) Corresponding Western blot of β-actin. Molecular weight markers are seen on the right of each blot: Precision plus protein unstained standards marker (left, Bio-Rad), Spectra Multicolor Broad Range Protein Ladder pre-stained marker (right, Thermo Fisher Scientific).
